# AuNPs-Based Thermoresponsive Nanoreactor as an Efficient Catalyst for the Reduction of 4-Nitrophenol

**DOI:** 10.3390/nano8120963

**Published:** 2018-11-22

**Authors:** Wei Liu, Xiaolian Zhu, Chengcheng Xu, Zhao Dai, Zhaohui Meng

**Affiliations:** 1 State Key Laboratory of Separation Membranes and Membrane Processes/National Center for International Joint Research on Separation Membranes, Tianjin Polytechnic University, Tianjin 300387, China; 2 School of Environmental and Chemical Engineering, Tianjin Polytechnic University, Tianjin 300387, China; zxl280060217@163.com (X.Z.); 13820360661@163.com (C.X.); 3 Tianjin Colouroad Coatings & Chemicals Co Ltd, Tianjin 300457, China; colouroadmzh@163.com

**Keywords:** nanoreactor, distillation precipitation polymerization, catalytic reduction, thermosensitive, AuNPs

## Abstract

A new AuNPs-based thermosensitive nanoreactor (SiO_2_@PMBA@Au@PNIPAM) was designed and prepared by stabilizing AuNPs in the layer of poly(N,N’-methylenebisacrylamide) (PMBA) and subsequent wrapping with the temperature-sensitive poly(N-isopropylacrylamide) (PNIPAM) layer. The new nanoreactor exhibited high dispersibility and stability in aqueous solution and effectively prevented the aggregation of AuNPs caused by the phase transformation of PNIPAM. The XPS and ATR-FTIR results indicated that AuNPs could be well stabilized by PMBA due to the electron transfer between the N atoms of amide groups in the PMBA and Au atoms of AuNPs. The catalytic activity and thermoresponsive property of the new nanoreactor were invested by the reduction of the environmental pollutant, 4-nitrophenol (4-NP), with NaBH_4_ as a reductant. It exhibited a higher catalytic activity at 20 °C and 30 °C (below LCST of PNIPAM), but an inhibited catalytic activity at 40 °C (above LCST of PNIPAM). The PNIPAM layer played a switching role in controlling the catalytic rate by altering the reaction temperature. In addition, this nanoreactor showed an easily recyclable property due to the existence of a silica core and also preserved a rather high catalytic efficiency after 16 times of recycling.

## 1. Introduction

Gold nanoparticles (AuNPs) have been extensively discussed and used in a variety of areas, such as the catalysis [1], drug delivery [2], biomedical therapy [3], and environmental detection [4], due to their superior properties in the physical, chemical, and biological aspects. However, because of their colloidal nature and higher surface energies, AuNPs tended to aggregate during the synthesis process, which would cause great limitations in the application of their excellent properties. Therefore, improving the stability and monodispersity of AuNPs could be an important step to increase their efficiency in practical applications.

Generally, AuNPs could be stabilized by polymers [5], ligand [6], surfactant [7], and so on. The typical stabilizers of AuNPs are thiol or dithio ligands [8,9]. However, the catalytic activity of AuNPs would decrease significantly due to the strong interaction between the thiol or dithio groups and AuNPs [10], which hindered its application in the catalysis. Polymers, as a perfectly protected shell for AuNPs, have attracted extensive attention in the research and industrial fields because they have different swollen and flexible matrix structures for easy permeation of the reactants [11]. However, among the polymer matrices of the protected AuNPs, a majority of them only act as supports to the AuNPs, which results in limitations of their potential applications. AuNPs stabilized by stimuli-responsive polymers have opened a new window for the development of future smart materials. Poly(N-isopropylacrylamide) (PNIPAM), as a typical thermoresponsive polymer, has been used as the stabilizer for AuNPs because of their amphiphilic nature, unique network structure, and coordinative interaction between Au atoms and ligand heteroatoms of PNIPAM.

In previous publications, most of researchers have reported on the AuNPs encapsulated directly by PNIPAM. For instance, Maji et al. [12] prepared thermosensitive gold nanoparticles coated by poly(N-isopropylacrylamide) and used them as the colourimetric temperature and salt sensors. However, due to the reversible phase transition of PNIPAM in aqueous solution at the temperature below and above the lower critical solution temperature (LCST) of PNIPAM, the stability of the inner AuNPs would be affected and they could aggregate during the preparation and using process, which would greatly restrict their application in other aspects. The aggregation behavior of AuNPs could be evidenced from the color transition of a PNIPAM-AuNPs aqueous solution below and above the LCST. When heating to the above LCST, the extended PNIPAM chains collapsed, causing AuNPs aggregation and the solution turned from red to blue [13]. Chakraborty et al. [14] reported the color changes of PNIPAM-modified AuNPs after 1 h of heating these particles to 40 °C. Therefore, designing an appropriate nanoreactor structure that could not only combine gold nanoparticles with PNIPAM, but also ensure the stability of AuNPs remains a challenge for the development of new nanomaterials.

In this work, we reported a novel nanoreactor (SiO_2_@PMBA@Au@PNIPAM) with high stability for AuNPs and excellent thermosensitivity by stabilizing AuNPs in the gel layer of PMBA and then wrapping AuNPs with PNIPAM. Herein, the inner SiO_2_ core was synthesized by the traditional stӧber method, which could be used as the template to ensure the regular shape of the synthesized novel nanoreactor and increase the specific gravity for the convenient centrifugalization and reutilization. PMBA acts as a good stabilizer for AuNPs and makes it easy to wrap on the surface of the silica core by distillation-precipitation polymerization. AuNPs was immobilized on the layer of PMBA by *in-situ* reduction of gold precursors, HAuCl_4_. PNIPAM, acting as a thermosensitive protected-shell, can be used as a “switch” to control the contact between the external small molecules and the internal AuNPs by altering the temperature.

A nanoreactor is a kind of important reactor in the chemical transformation, which can complete the chemical conversion in a confined space of the nano scale. The nanoreactor has great application potential in the catalysis field, in particular for thermosensitive “smart nanoreactors”, which could regulate the catalytic rate by altering the temperature of catalytic systems. For instance, Satapathy et al. [15] successfully synthesized a series of hybrid-nanostructured PNIPAM-Au with different shapes of AuNPs for the reduction of 4-nitrophenol (4-NP), and observed a controllable catalytic activity by altering the reaction temperature. Chen et al. [16] designed a new nanoreactor structure with the spatial separation of Au and PNIPAM to use as the catalyst to reduce 4-NP, which exhibited obviously different catalytic activity at the temperature below and above the LCST of PNIPAM.

4-nitrophenol (4-NP) was considered as one of the most difficult organic pollutants to decompose because of the water solubility, excellent chemical properties, and biological stability, and was listed as the priority toxic pollutants by the United States Environmental Protection Agency (USEPA) [17,18,19,20]. Moreover, the reductive product, 4-aminophenol (4-AP), was an important chemical and pharmaceutical intermediate, and has wide application in the industrial and medical fields [21,22]. Performing the catalytic reduction of 4-nitrophenol (4-NP) to 4-aminophenol (4-AP) as a model experiment was not only an ideal system to study the catalytic activity and thermosensitivity of the newly synthesized catalysts, but also an efficient method for the removal of 4-NP from the aquatic environment.

In this work, we further chose the catalytic reduction of 4-NP to 4-AP, with NaBH_4_ as the reductant, as the model experiment to study the catalytic ability and thermoresponsive property of the new nanoreactor. Here, the AuNPs stabilized by PMBA acted as the catalysts to trigger the reduction reaction. The color of the novel nanoreactor aqueous solution did not change even upon heating to 40 °C for 2 h, which indicated that AuNPs in the novel reactor did not aggregate in the process of the phase transformation of PNIPAM. In order to study the temperature sensitive properties of the novel reactor, the reduction of 4-NP was performed at 20 °C, 30 °C (below LCST of PNIPAM), and 40 °C (above LCST of PNIPAM), respectively, where the novel nanoreactor has shown a higher catalytic activity at 20 °C and 30 °C, but an inhibited catalytic activity at 40 °C. Moreover, the new nanoreactor showed a good cycling performance, while still preserving higher catalytic efficiency even after 16 times of recycling.

## 2. Materials and Methods

### 2.1. Materials

Acetonitrile (analytical grade, Tianjin Concord Technology Co., Ltd., Tianjin, China) was dried over calcium hydride and purified by distillation before use. Tetraethyl orthosilicate (TEOS, analytical grade), 3-(trimethoxysilyl) propylmethacrylate (MPS, analytical grade), and (3-aminopropyl) triethoxysilane (APTES, 99%) were all used as purchased from Aladdin Industrial Corporation (Shanghai, China). N,N’-Methylenebisacrylamide (MBA, 97%, Aladdin Industrial Corporation) was recrystallized from ethanol. 2,2’-Azobisisobutyronitrile (AIBN, Aladdin Industrial Corporation) was recrystallized from methanol. N-Isopropylacrylamide (NIPAM, analytical grade, Aladdin Industrial Corporation) was recrystallized from hexane. Sodium borohydride (NaBH_4_, analytical grade) was obtained from Tianjin Fengchuan Chemical reagent Technologles Co., Ltd. (Tianjin, China). 4-Nitrophenol (4-NP, analytical grade) was taken from Tianjin Krem chemical reagents Co., Ltd. (Tianjin, China). Chlorchloric acid solution (HAuCl_4_) was prepared from analytical grade AuCl_3_∙HCl∙4H_2_O (Aladdin Industrial Corporation) using acetonitrile after distillation.

### 2.2. Synthesis

#### 2.2.1. Preparation of MPS-Modified SiO_2_ Microspheres

SiO_2_ microspheres with a diameter of 70 nm were prepared by a classical Stöber method [23] and the surface was modified by MPS. A typical procedure for the preparation of SiO_2_ microspheres by the modified Stöber method was given as follows. 50 mL ethanol, 1 mL deionized water, and 1.8 mL NH_3_·H_2_O were added into a 100 mL round-bottomed flask and stirred violently for 20 min. Then, 1.2 mL of TEOS was added into the solution, and the resulting mixture was stirred for 5 h. After that, 0.07 mL of MPS was put into the mixture to introduce the double bonds on the surface of SiO_2_ microspheres over a reaction period of 19 h. After the reaction, the resultant SiO_2_ microspheres with double bonds (SiO_2_–C=C) were separated by centrifugation, then dispersed in ethanol, and centrifuged again. The products were washed for four times in this way and the final products were dispersed in 30 mL of acetonitrile for the next step.

#### 2.2.2. Preparation of SiO_2_@PMBA Core-Shell Microspheres

The surface of SiO_2_ microspheres was too smooth, which was not convenient to load and stabilize Au nanoparticles. Therefore, we wrapped a layer of PMBA on the surface of the silica microspheres (SiO_2_@PMBA). A typical procedure for the polymerization was as follows. MPS-modified SiO_2_ microspheres (as-produced in Section 2.2.1, 0.3 g·L^−1^ of 5 mL acetonitrile) were dispersed in 35 mL of acetonitrile at room temperature, marked as mixture A. 0.05 g of MBA was dispersed in 20 mL of acetonitrile, marked as mixture B. AIBN (0.001 g, 2 wt% relative to the monomer) was dispersed in 20 mL of acetonitrile, marked as mixture C. Then, mixture A, mixture B, and mixture C were added into a 100-mL round-bottomed flask equipped with a fractionating column, Liebig condenser, and a receiver. The flask was submerged in a mantle with a magnet. The reaction was stirred in 400 rpm and heated from the room temperature to a boiling state within 12 min, and the reaction mixture became milky white after boiling for 2 min. The reaction system was kept under the refluxing state for 8 min and the acetonitrile was then distilled out from the reaction system. The reaction was stopped until distilling 17 mL of acetonitrile within 1 h. After the reaction, the resultant SiO_2_@PMBA core-shell microspheres were separated by centrifugation, then dispersed in acetonitrile, and centrifuged again. The products were washed for four times in this way and the final products were dispersed in 30 mL of acetonitrile for the next step.

#### 2.2.3. Preparation of SiO_2_@PMBA@Au microspheres

AuNPs were stabilized on the PMBA polymer shell layer of SiO_2_@PMBA by *in-situ* reduction of AuCl_4_^−^ in the presence of SiO_2_@PMBA core-shell microspheres. A typical procedure of the *in-situ* reduction was as follows. The SiO_2_@PMBA microspheres (as-produced in Section 2.2.2, 0.34 g·L^−1^ of 15 mL acetonitrile) were dispersed in 60 mL of acetonitrile. Then, 0.7 mL of HAuCl_4_ (0.024 mol·L^−1^) acetonitrile solution was added into the suspension, followed by dispersing for 5 min with ultrasonic, and then stirring for 12 h. After that, 0.05 mL of 0.51 mol·L^−1^ NaBH_4_ was added into the mixture, accompanied with the color of the mixture gradually changing from light yellow to purple red. After being agitated for 1 h, the resultant SiO_2_@PMBA@Au microspheres were separated by centrifugation, then dispersed in acetonitrile, and centrifuged again. The products were washed for four times in this way and the final products were dispersed in 30 mL of acetonitrile for the next step.

#### 2.2.4. Preparation of Thermosensitive SiO_2_@PMBA@Au@PNIPAM Microspheres

In order to introduce the thermosensitive properties, a PNIPAM outer shell for coating the SiO_2_@PMBA@Au microspheres were synthesized in the presence of SiO_2_@PMBA@Au microspheres as the template by seeding distillation precipitation polymerization [24] with MBA as the crosslinker, NIPAM as the functional monomer, AIBN as the initiator, and acetonitrile as the solvent (SiO_2_@PMBA@Au@PNIPAM). In the standard recipe, the SiO_2_@PMBA@Au microspheres (as-produced in Section 2.2.3, 0.35 g·L^−1^ of 15 mL acetonitrile) were dispersed in 25 mL of acetonitrile, marked as mixture A. 0.05 g of MBA and 0.05 g of NIPAM were dispersed in 20 mL of acetonitrile, marked as mixture B. AIBN (0.002 g, 2 wt% relative to the monomer) was dispersed in 20 mL of acetonitrile, marked as mixture C. Then, mixture A, mixture B, and mixture C were simultaneously added into a 100-mL round-bottomed flask, attached with a fractionating column, Liebig condenser, and a receiver. The flask was submerged in a mantle with a magnet. The reaction was stirred in 400 rpm and heated from room temperature to a boiling state within 13 min. Then, the solvent was distilled out from the reaction system for about 18 min and the reaction was stopped after 14 mL of the acetonitrile was distilled from the reaction system within 50 min. After the polymerization, the resulting thermosensitive SiO_2_@PMBA@Au@PNIPAM microspheres were separated by centrifugation, then dispersed in acetonitrile, and centrifuged again. The products were washed for four times in this way and the final products were dried in a vacuum oven at 60 °C to constant weight, and afforded 0.095 g products with the yield of 90.3%.

### 2.3. Characterization

The size and morphology of the thermosensitive SiO_2_@PMBA@Au@PNIPAM microspheres were determined by transmission electron microscopy (TEM) using a Hitachi-7650 microscope (Hitachi Limited, Tokyo, Japan), which was performed at an acceleration voltage of 100 kV and a current of 10 A. The samples for TEM characterization were dispersed in acetonitrile and dropped onto the surface of a copper grid coated with a carbon membrane and then dried in a vacuum oven at the room temperature. All the size and size distribution reflected the average over 100 particles, which were calculated by the following formulae:

(1) $$U=D_{w}/D_{n}\;D_{n}=\sum_{i}^{k}n_{i}D_{i}/\sum_{i}^{k}n_{i}\;D_{w}=\sum_{i}^{k}n_{i}D_{i}^{4}/\sum_{i}^{k}n_{i}D_{i}^{3}$$

where U is the polydispersity index, D_n_ is the number-average diameter, D_w_ is the weight-average diameter, N is the total number of the measured particles, and D_i_ is the diameter of the determined microsphere.

The elemental analysis of the SiO_2_@PMBA@Au@PNIPAM microspheres was applied by the energy-dispersive X-ray spectroscopy (EDX), EDX mapping, and scanning transmission electron microscopy (STEM) equipped with transmission electron microscopy (TEM) (TecnaiG^2^ F20, FEI, Hillsboro, OR, USA).

The interaction nature between AuNPs and functional groups on microspheres was analyzed by X-ray photoelectron spectroscopy (XPS) with a standard AlKa excitation source (*hv* =1486.6 eV, K-alpha, Thermofisher, Waltham, MA, USA).

^1^H-NMR spectra of the SiO_2_@PMBA microspheres and SiO_2_@PMBA@Au microspheres in D_2_O was measured by a Bruker 400M Nuclear Magnetic Resonance (NMR) spectrometer (Billerica, MA, USA).

Attenuated Total Reflectance (ATR)-Fourier Transformed Infrared (FTIR) spectroscopy was recorded on a Thermo Nicolet 6700 FT-IR spectrometer (Waltham, MA, USA) from 4000 to 400 cm^−1^, equipped with ATR single reflection unit mounting a diamond crystal. The absorption spectra were collected by averaging 32 scans with a resolution of 2 cm^−1^.

The loading capacity of the gold on SiO_2_@PMBA microspheres was measured by atomic absorption spectrometry (AAS) (contrAA 700, Analytik Jena AG, Jena, Germany).

### 2.4. Catalytic Activity

The catalytic properties of the thermosensitive SiO_2_@PMBA@Au@PNIPAM microspheres was investigated by the catalytic reduction of 4-NP to 4-AP with NaBH_4_ solution as the reductant. A typical procedure for the catalytic reduction of 4-NP was as follows: 0.15 g of NaBH_4_ was added into the 100 mL of the 0.088 mmol·L^−1^ 4-NP aqueous solution and the mixture was stirred for 10 min. Then, 0.018 g of the thermosensitive SiO_2_@PMBA@Au@PNIPAM microspheres as the catalyst was added into the 100 mL of the mixture to initiate the reduction reaction. In order to monitor the kinetics of the reaction constantly, 1 mL of samples were taken out from the reaction system every two minutes, and immediately separated by the centrifuge. The supernatant was determined via UV-vis spectroscopy by recording the absorption at 400 nm at ambient temperature (20 °C). The whole reaction process was ended until the yellow color of the solution gradually disappeared. The same catalytic reduction experiments were carried out at 30 °C and 40 °C, respectively, to investigate the thermodynamics of the reaction.

To perform the recoverable catalytic activity of the thermosensitive SiO_2_@PMBA@Au@PNIPAM microspheres, we carried out the reusing experiment of the microspheres. The catalyst was separated by centrifugation after the complete reduction of 4-NP and redispersed in the next recycling reaction system for subsequent catalytic experiments under the same reaction conditions. The process of catalytic reduction was repeated for 16 times by the same method.

## 3. Results and Discussion

**Scheme 1:**
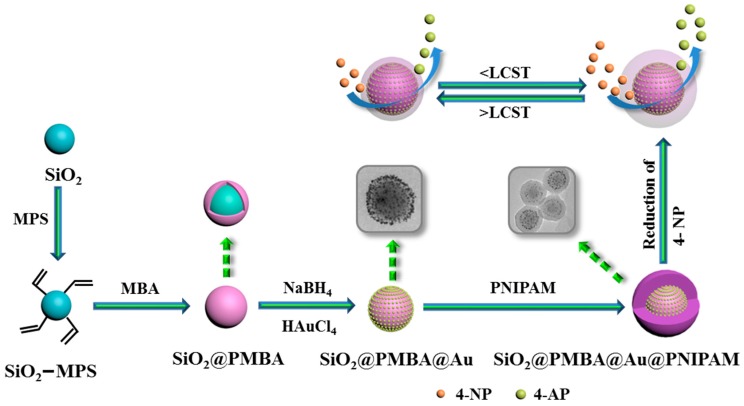
The synthesis procedure of the new nanoreactor and the application as a catalyst for the reduction of 4-nitrophenol (4-NP).

The synthetic procedure of the novel nanoreactor structure (SiO_2_@PMBA@Au@PNIPAM) and the application as a catalyst are illustrated briefly in Scheme 1. First of all, SiO_2_ microspheres were prepared by the traditional Stӧber method and then modified by MPS to introduce the double bonds on the surface of the microspheres, fabricating SiO_2_-MPS microspheres for the following polymerization. In order to stabilize AuNPs, a layer of PMBA was then capped on the surface of the SiO_2_-MPS microspheres by distillation precipitation polymerization. Subsequently, AuNPs were stabilized on the layer of the PMBA by *in-situ* reduction of HAuCl_4_ (i.e., SiO_2_@PMBA@Au). Afterwards, PNIPAM was wrapped on the as-synthesized SiO_2_@PMBA@Au microspheres by distillation precipitation polymerization to import the thermosensitive property, forming the new nanoreactor, SiO_2_@PMBA@Au@PNIPAM. Finally, the catalytic activity and thermoresponsive property of the new nanoreactor were invested by the reduction of the environmental pollutant, 4-NP. The reduction processing was applied at two different temperatures, below and above the LCST, respectively. When the reaction temperature was below the LCST of PNIPAM, the PNIPAM out layer of the as-synthesized nanoreactor showed a state of expansion, and the nanoreactor exhibited a higher catalytic activity for 4-NP. In contrast, when the reaction temperature was above the LCST of PNIPAM, the PNIPAM layer collapsed and shrunk, which would decrease the diffusion rate of the 4-NP molecules through the PNIPAM layer, and inhibit the catalytic activity of the nanoreactor.

### 3.1. Preparation and Characterization of the SiO_2_@PMBA@Au@PNIPAM Microspheres

**Figure 1:**
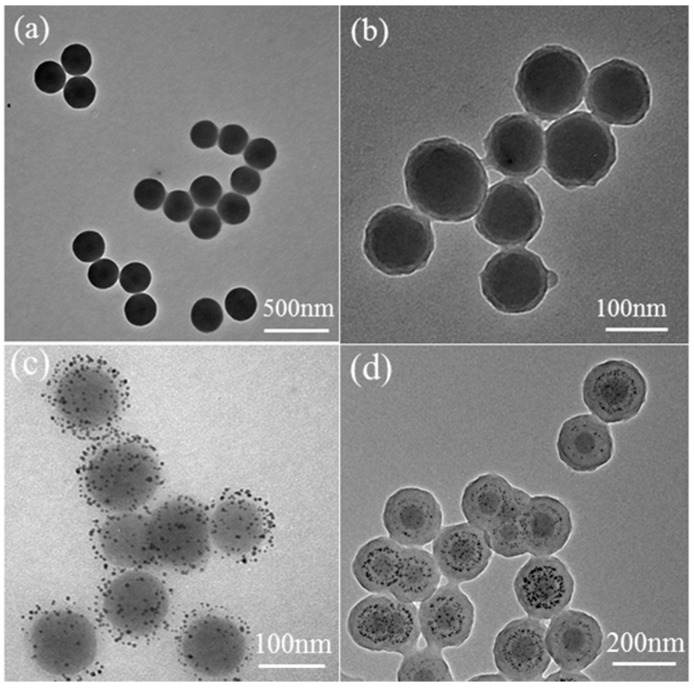
TEM micrographs of synthesized microspheres: (**a**) SiO_2_-MPS silica microspheres; (**b**) SiO_2_@PMBA microspheres; (**c**) SiO_2_@PMBA@Au microspheres; (**d**) SiO_2_@PMBA@Au@PNIPAM microspheres.

**Figure 2:**
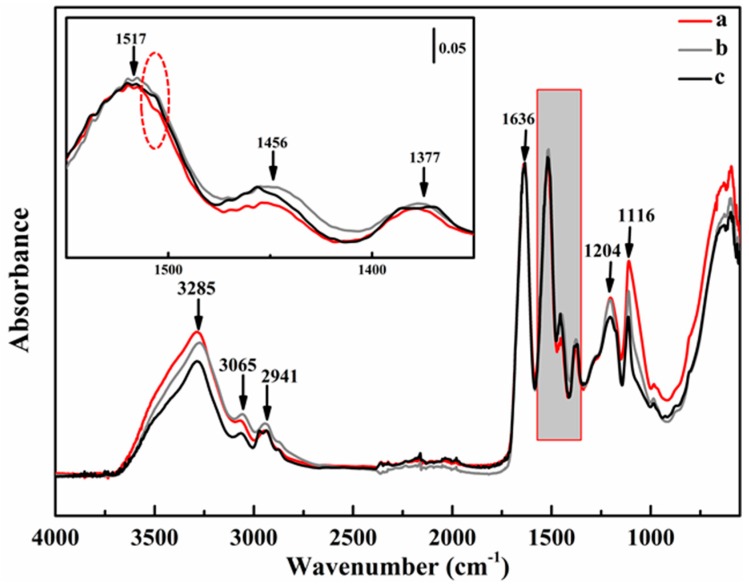
Attenuated total reflectance-fourier transformed infrared (ATR-FTIR) spectra of the synthesized microspheres: (**a**) SiO_2_@PMBA, (**b**) SiO_2_@PMBA@Au, (**c**) SiO_2_@PMBA@Au@PNIPAM.

In this work, the new thermosensitive nanoreactor was prepared in four steps as shown in the Experimental section. Here, the SiO_2_ microspheres were first synthetized by the traditional Stӧber method, and then further functionalized with a layer of MPS to introduce the C=C groups, which could generate free radicals in the presence of the initiator and trigger the polymerization of MBA to form a layer of PMBA at the external surface of the SiO_2_ microspheres (i.e., SiO_2_-MPS). The Stӧber method has been proved to be a widely used method for preparing monodisperse silica microspheres with very high yield, and the size of the silica microspheres can be controlled strictly by changing the amount of ammonium hydroxide [24,25]. Herein, SiO_2_-MPS microspheres with a diameter of 70 nm were used as the template of the new nanoreactor. The TEM image of the corresponding SiO_2_-MPS microspheres is shown in Figure 1a, which exhibited a regular spherical shape with a smooth surface and a uniform size of 70 nm. In order to stabilize AuNPs, PMBA was selected as a stabilizer and coated on the surface of the SiO_2_-MPS microspheres by distillation precipitation polymerization (i.e., SiO_2_@PMBA). The formation of the layer of PMBA was confirmed by TEM as shown in Figure 1b. It was obvious that PMBA was uniformly coated on the surface of SiO_2_-MPS microspheres, forming a well-defined core-shell structure. Compared with the SiO_2_-MPS microspheres in Figure 1a, gel-like coating layers were observed in the TEM image of SiO_2_@PMBA because of different mass contrasts between the inorganic core and polymeric shell domain. The diameters of the SiO_2_@PMBA microspheres were 28 nm larger than those of the SiO_2_-MPS microspheres, which indicated that the thickness of the PMBA layer was 14 nm. In addition, the thickness could be readily controlled by adjusting the amount of the MBA monomer and the reaction time during polymerization. The presence of PMBA on the surface of SiO_2_-MPS microspheres was further confirmed by ATR-FTIR, as shown in Figure 2a. The strong peaks at 1636 cm^−1^ and 1517 cm^−1^ were, respectively, attributed to the characteristic vibration of υ(C=O) and υ(C–N) of the amide groups of PMBA, indicating that PMBA was successfully coated on the surface of SiO_2_-MPS microspheres.

**Figure 3:**
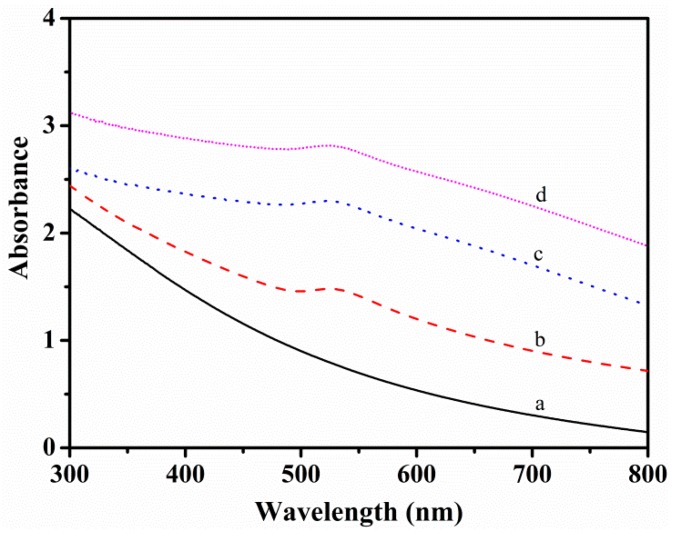
UV-vis absorption spectra of aqueous dispersions for synthesized microspheres: (**a**) SiO_2_@PMBA microspheres; (**b**) SiO_2_@PMBA@Au microspheres; (**c**) SiO_2_@PMBA@Au@PNIPAM microspheres (20 °C); (**d**) SiO_2_@PMBA@Au@PNIPAM microspheres (40 °C).

AuNPs with large specific surface areas and abundant active surface atoms demonstrated excellent catalytic activities in the catalytic field, so we synthetized the new reactor structure equipped with AuNPs [11]. Figure 1c showed the TEM image after stabilizing AuNPs on the gel layer of PMBA (i.e., SiO_2_@PMBA@Au). It was obvious that AuNPs corresponding to the small black spots in the image were embedded within the gel layer of PMBA with the low contrast. The embedded AuNPs were very stable without much aggregation, which indicated that PMBA could be used as a good stabilizer for stabilizing AuNPs. The average diameter of the AuNPs was about 7 nm and the polydispersity index (U) reflecting the size distribution of these particles was 1.004, which was lower than 1.01 and indicated that the AuNPs were highly monodisperse. The surface plasmon band of AuNPs is known to be sensitive to the size of the particles and their surrounding environment [13]. As shown in Figure 3a,b, the aqueous solution of highly dispersed SiO_2_@PMBA microspheres does not exhibit any absorption whereas a new absorbance band appeared at 523 nm due to the surface plasmon resonance of AuNPs after stabilizing Au on the gel layer of SiO_2_@PMBA. The result also indicated that the sizes of the AuNPs were below 10 nm, which was consistent with the report of Wu et al [26]. The loading capacity of the gold on SiO_2_@PMBA microspheres was 5.67% (2.88 × 10^−2^ mmoL/g) as measured by AAS. In order to make the nanoreactor temperature-sensitive and realize the intelligent control of the catalytic rate, NIPAM as a thermosensitive monomer was wrapped on the outermost layer of SiO_2_@PMBA@Au (i.e., SiO_2_@PMBA@Au@PNIPAM). As shown in Figure 1d, the SiO_2_@PMBA@Au@PNIPAM microspheres with the spherical shape, clear hierarchical structure, and uniform size were presented. A light gray outer ring corresponding to PNIPAM was observed on the surface of all the SiO_2_@PMBA@Au microspheres, indicating that the coating of PNIPAM was quite uniform. The ATR-FTIR spectra of SiO_2_@PMBA@Au@PNIPAM in Figure 2c provided powerful evidence of successful coating of PNIPAM. In addition to the characteristic peaks of the amide groups mentioned above, two doublet peaks appeared at 1377 cm^−1^, and 2941 cm^−1^, which were assigned to δ((–CH_3_)_2_CH–) and υ_as_(–CH_2_) of PNIPAM, respectively. Figure 3c,d shows the UV-vis absorption spectra of the aqueous solution with the highly dispersed SiO_2_@PMBA@Au@PNIPAM microspheres both at room temperature (20 °C) and above the LCST of PNIPAM (40 °C). The maxima of the surface plasmon absorption at two temperatures were all located at 523 nm with no shift, which indicated that the AuNPs in the gel layer of PMBA did not aggregate and thus proved that the stability of AuNPs were not affected by the phase transition of PNIPAM.

**Figure 4:**
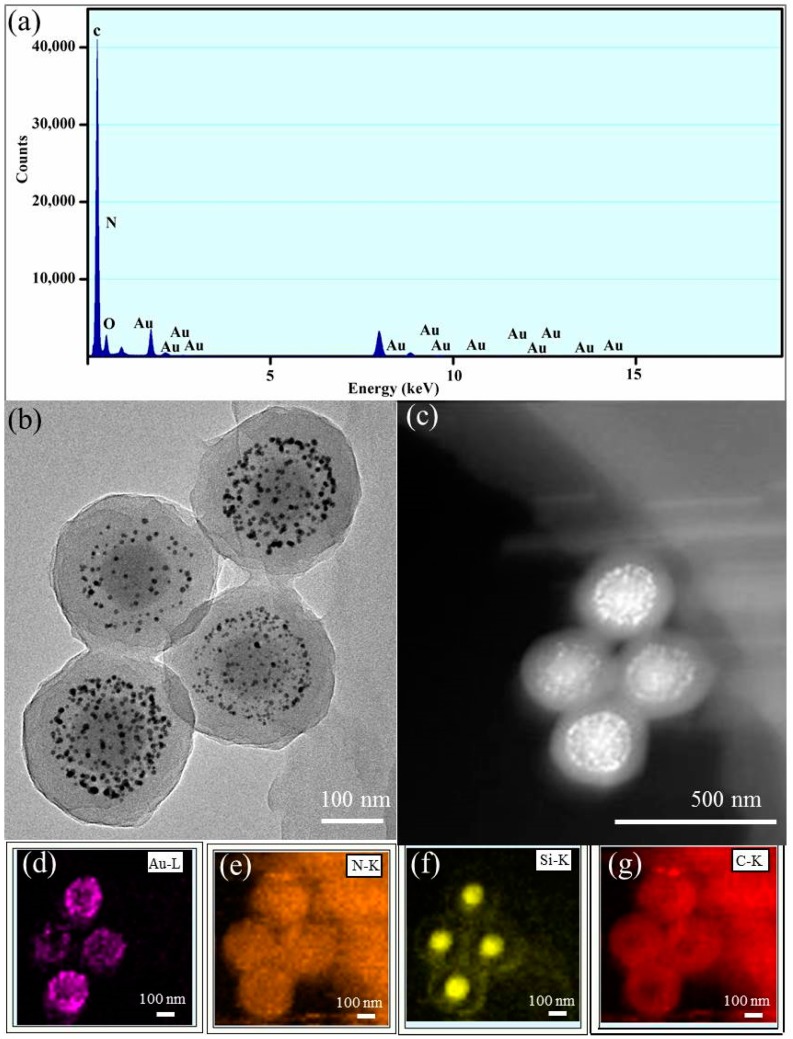
Characterization of the SiO_2_@PMBA@Au@PNIPAM microspheres: (**a**) spectrum of Energy-dispersive X-ray spectroscopy (EDX); (**b**) image of Transmission electron microscopy (TEM); (**c**) image of Scanning transmission electron microscopy (STEM); (**d**)–(**g**) EDX mapping of the SiO_2_@PMBA@Au@PNIPAM microsphere in (**c**) with the Au, N, Si, and C elements, respectively.

The elements and their distributions of the new nanoreactor were further investigated by STEM and EDX. In Figure 4a, one can clearly observe that the SiO_2_@PMBA@Au@PNIPAM microspheres were composed of C, N, O, Si, and Au elements. The elemental mapping in Figure 4d–g revealed that C, N, and Au were homogenously distributed within the new nanoreactor while the distribution area of Au was smaller than that of C and N due to the PNIPAM layer. Silica microspheres as the core were located in the middle of the new nanoreactor and the distribution area was the smallest, which was consistent with the TEM images. Figure 4c showed the STEM image of the SiO_2_@PMBA@Au@PNIPAM. It was obvious that the SiO_2_@PMBA@Au@PNIPAM microsphere was made up of two parts with different brightness, in which the inner bright part corresponded to SiO_2_@PMBA@Au, and the external blurred part corresponded to the PNIPAM layer. Moreover, the thickness of the external blurred part was about 19 nm, which was consistent with the result of the TEM characterization in Figure 4b. All of these results confirmed the new nanoreactor structure had been successfully synthesized.

### 3.2. The Interaction Nature Between Au NPs and Functional Groups in the New Nanoreactor

In the present work, the TEM image results indicated that AuNPs can be well stabilized in the new nanoreactor. In order to invest the interaction mechanism of the functional groups and AuNPs in the new nanoreactor, the FTIR spectra of three microspheres (SiO_2_@PMBA, SiO_2_@PMBA@Au, and SiO_2_@PMBA@Au@PNIPAM) were collected. Here, the functional groups were the amide groups introduced by MBA and NIPAM monomers, so we focused on the absorption peaks of amide groups in the later analysis. In Figure 2, the characteristic amide I, II, and III bands representing amide groups were observed at 1636 cm^−1^, 1517 cm^−1^, and 1204 cm^−1^, which corresponded to υ(C=O), υ(C–N), and υ(CNH), respectively. In addition, the overtone band of 1517 cm^−1^ also appeared at 3065 cm^−1^, further proving the existence of the C–N bond. The strong band at 3285 cm^−1^ was assigned to the vibration mode of υ(NH). By comparing the absorption bands of amide groups in three microspheres, one can observe that a new shoulder peak appeared at the lower frequency of 1515 cm^−1^ for SiO_2_@PMBA@Au and SiO_2_@PMBA@Au@PNIPAM, as shown in the circle part of the local enlarged drawing in the upper-left corner, which was due to the complicated interaction between the C–N amide group and AuNPs, confirming the formation of a coordination bond between the Au atoms and the N atoms in the amide groups.

**Figure 5:**
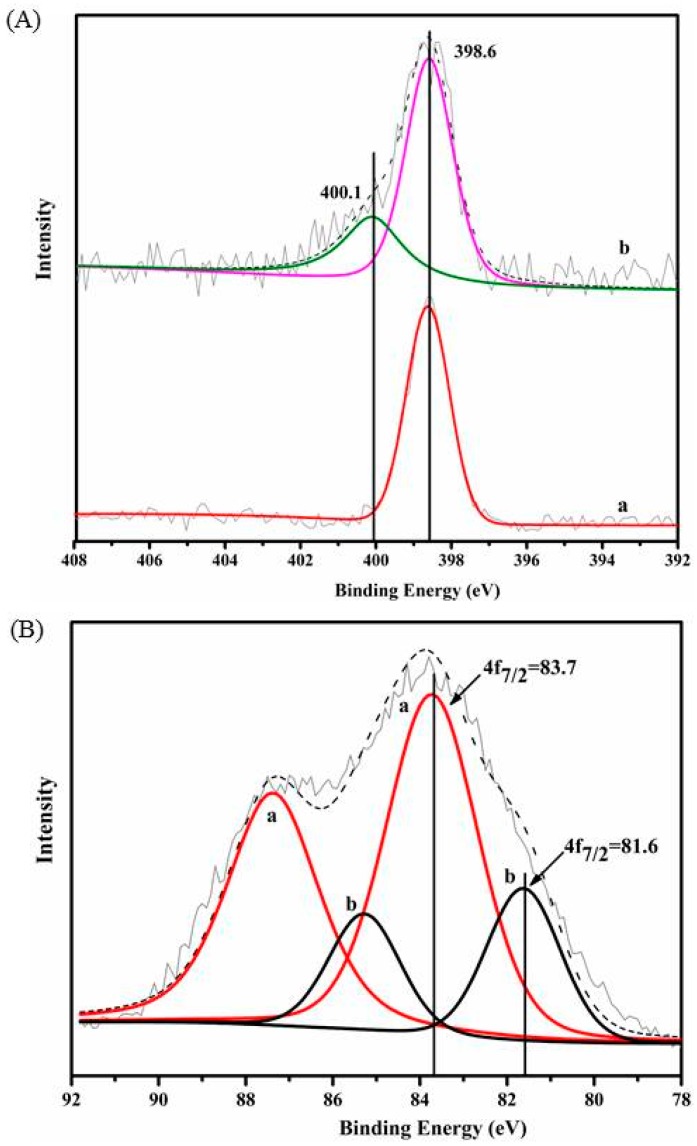
X-ray photoelectron spectroscopy (XPS) of the N 1*s* and Au 4*f* for the synthesized microspheres: (**A**) Group a is the binding energy (BE) of N 1*s* for SiO_2_@PMBA; group b is the BE of N 1*s* for SiO_2_@PMBA@Au; (**B**) BE of Au 4*f* for the SiO_2_@PMBA@Au microspheres. Here, a and b are two groups of Au 4*f* distinct peaks for Au atoms with different chemical environments.

To further understand the interaction nature between the amide groups and AuNPs, XPS of Au 4*f* and N 1*s* were measured for the as-synthesized microspheres as shown in Figure 5. XPS is an effective method for analyzing the interaction at the interface, in particular for the solid material, which has been extensively used to investigate the interaction between the stabilizer and Au particles [27,28]. Figure 5A showed the XPS spectra of N 1*s* for SiO_2_@PMBA microspheres (curve a) and SiO_2_@PMBA@Au microspheres (curve b), respectively. In curve a, the peak at 398.6 eV corresponded to the N 1*s* of amide for SiO_2_@PMBA; while in curve b, two peaks at 400.1 eV and 398.6 eV were found, which represented the N 1*s* of amide for SiO_2_@PMBA@Au in two different chemical environments. The large peak at 398.6 eV reflected the majority of the N atoms in PMBA; the small peak at 400.1 eV corresponded to the N atoms in the state of lower electronic density, which provided evidence for the N atoms donating their electrons to the Au atoms after immobilizing AuNPs on the SiO_2_@PMBA microspheres. For SiO_2_@PMBA@Au, XPS spectra of the Au 4*f* core level in Figure 5B showed two groups of peaks, illustrated as group a and group b, respectively. The appearance of these two groups of peaks exhibited that the AuNPs were under two kinds of chemical environments [29]. According to the Au 4*f*_7/2_ core level, one peak was located at 83.7 eV (in group a) and the other was at 81.6 eV (in group b). Both of the two peaks for Au 4*f*_7/2_ were lower than that of the metallic Au (84.0 eV) [30], which indicated that the AuNPs of SiO_2_@PMBA@Au had accepted electrons from N atoms of amide groups. The results were consistent with the discussion about N 1*s* just mentioned above. The peak at 83.7 eV was bigger than the other one at the lower BE. Since the average size of AuNPs was 7 nm in this case, most of the gold atoms were located in the interior of AuNPs, which produced much weaker interactions with N atoms, resulting in a lower BE shift of only 0.3 eV (from 84.0 eV to 83.7 eV) as shown in group a. In comparison, a small number of gold atoms were distributed on the surface of AuNPs, and their interaction with N atoms was stronger, corresponding to a larger BE shift of 2.4 eV (from 84.0 to 81.6 eV) as shown in group b. Therefore, the electron transfer between N atoms of amides and Au atoms of AuNPs determined the interaction nature between the amide groups and AuNPs, which could effectively prevent the aggregation of AuNPs during the formation process, and stabilize the AuNPs for storage and future utilization. In addition, the interaction nature between Au and N on SiO_2_@PMBA@Au microspheres was further proved by ^1^H-NMR spectra as shown in Figure S1 (Supporting Information). It was obvious that the sharp signal at 8.29 ppm of the amine proton in the amide groups completely flattened out after immobilizing AuNPs on the SiO_2_@PMBA microspheres, which indicated that AuNPs coordinated with N of the amide groups. The result was consistent with the change trend of NMR spectra for thiol ligands stabilized AuNPs [8].

### 3.3. Catalytic Activity and Thermoresponsive Property of the New Nanoreactor

**Figure 6:**
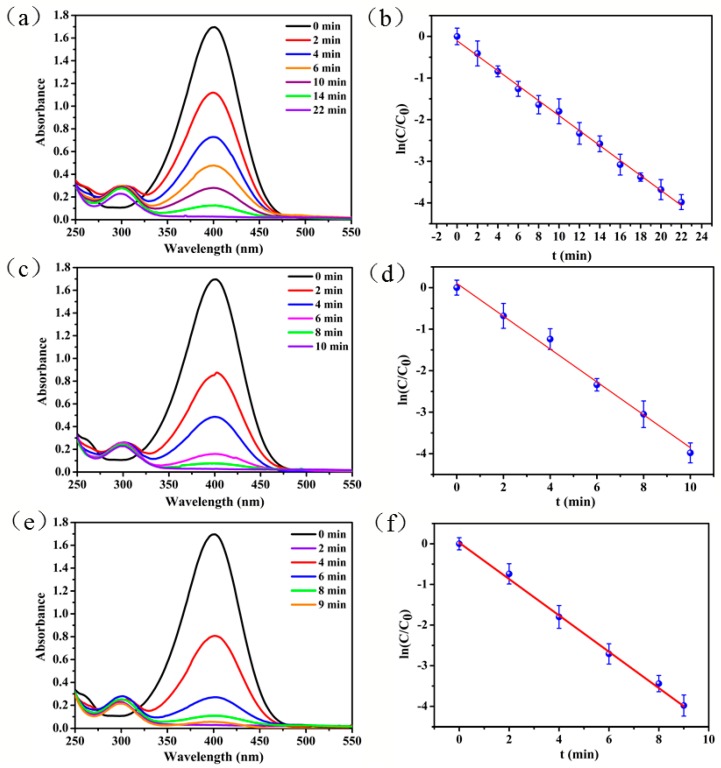
Time-dependent UV-vis spectra of the catalytic reduction of 4-NP to 4-AP and the corresponding plots of ln (C_t_/Co) versus time at different temperatures: 20 °C (**a**,**b**), 30 °C (**c**,**d**), and 40 °C (**e**,**f**).

It is well known that AuNPs are an excellent catalyst with high activity and selectivity, which have been widely applied in the catalytic fields [31,32]. In this work, the AuNPs were embedded in the gel layer of PMBA to prevent them from scattering or aggregation and wrapped by PNIPAM on the outer layer to introduce the temperature-sensitive characteristic for the new nanoreactor. In order to test the catalytic ability and thermoresponsive property, the reduction of 4-NP to 4-AP with NaBH_4_ as the reductant was used as the model reaction for the new nanoreactor. An aqueous solution of 4-NP in the presence of excess NaBH_4_ has a distinct UV-vis spectral profile with the maximal absorption at 400 nm, while the maximal absorption of 4-AP aqueous solution in the same condition was at 300 nm [28]. When the catalyst was added into the solution, the yellow color of 4-NP faded gradually together with simultaneous decreasing of the absorption peak at 400 nm. The whole catalytic process could be monitored by the UV-vis spectroscopy. Figure 6a showed the direct observation of UV-vis spectra for the catalytic reduction of 4-NP under ambient temperature (20 °C) using the new nanoreactor as catalysts in the presence of excess NaBH_4_. As the reaction was processing, the absorption peak at 400 nm decreased gradually with the appearance of a new absorption peak at 300 nm, indicating the reduction of 4-NP and the formation of 4-AP. The complete disappearance of the absorption peak at 400 nm in the UV-vis spectra occurred at 22 min, corresponding to termination of the reaction. The kinetic plot of the reduction of 4-NP is illustrated in Figure 6b, in which the plot of ln (C_t/_C_o_) versus time displays a good linear dependence, indicating that the reductive reaction followed the first-order kinetics. The pseudo-first-order rate constant (k) was obtained from the slope of the linear fitting line and turned out to be 0.181 min^−1^, which indicated that the new nanoreactor had higher catalytic activity for the reduction of 4-NP. This value was comparable to a similar work [33] reported earlier, in which AuNPs were stabilized on the graphene oxide functionalized with tannic acid (0.188 min^−1^) and was higher than that in the catalysis of hybrid PNIPAM-gold gels (0.066 min^−1^) [34].

Reaction temperature would have significant impact on the activity of the catalyst, so the reduction of 4-NP at different temperatures was carried out, while the other conditions remained unchanged. Generally, the reaction rate would be accelerated as the temperature increases for a common catalyst. In Figure 6c,d, when the temperature increased from 20 °C to 30 °C, the reaction rate (k) was enhanced from 0.181min^−1^ to 0.402 min^−1^ and the whole catalytic reaction took place only within 10 min. The apparent activation energy (Ea) was roughly calculated from the Arrhenius equation to be about 59 kJ·mol^−1^. This value was close to those of the previous reported catalysts AuNPs [16]. Furthermore, as shown in Figure 6e,f, when increasing the reaction temperature to 40 °C, the complete reduction of 4-NP to 4-AP occurred within 9 min with a first-order rate constant of 0.445 min^−1^, which was similar to the reaction rate at 30 °C without increasing. According to the Arrhenius equation (Ea = 59 kJ·mol^−1^ as calculated above), the theoretical catalytic rate at 40 °C should be 0.847 min^−1^, which indicated that the catalytic activity of the new nanoreactor was inhibited at 40 °C. The change of the rate constant was in accordance with the phase transition of PNIPAM, which indicated that the outermost PNIPAM layer of the new reactor played an important role in controlling the catalytic activity of the new nanoreactor. PNIPAM is a temperature sensitive polymer with the LCST at about 32 °C, which will undergo a reversible LCST phase transition from a swollen state to a shrunk state when PNIPAM was heated across the LCST [24]. For the reaction temperature below the LCST of PNIPAM (20 °C and 30 °C in this work), the PNIPAM chain was in a state of the extended conformation, the reactants of 4-NP and NaBH_4_ could easily be in contact with the inner AuNPs through the PNIPAM layer, initiating the reduction reaction. However, when the reaction was carried out above the LCST of PNIPAM (40 °C in this work), the PNIPAM chain collapsed and formed a polymer layer covering the surface of AuNPs [16], which caused an obstacle for the entries of external reactants. Therefore, the catalytic activity of the reactor at 40 °C was lower than that of the theoretical one. The PNIPAM layer in the new nanoreactor existed as a switch, which could regulate the reaction rate by altering the temperature of catalytic systems [22]. In addition, when the new nanoreactor was heated to 40 °C for 2 h, there was no color change for the nanoreactor, which indicated that the AuNPs in the new nanoreactor did not aggregate.

**Figure 7:**
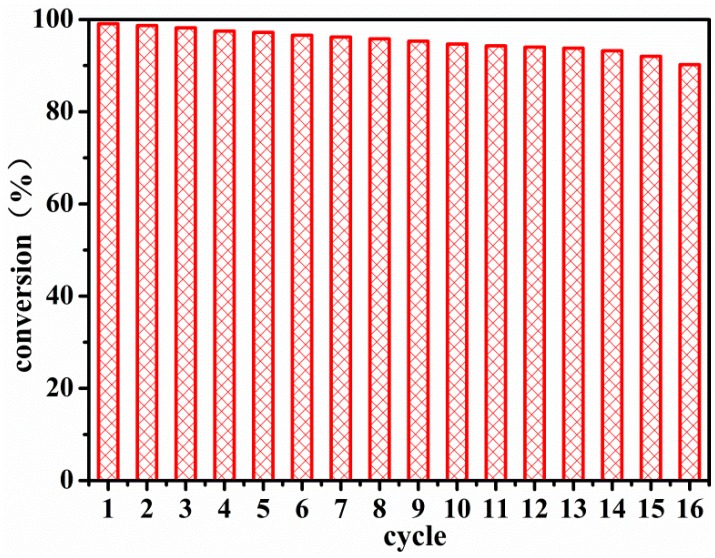
Reusability of the new nanoreactor as the catalyst for the reduction of 4-NP.

The stability and recyclability are particularly important for conventional catalysts. To investigate the reusability of the new nanoreactor as a recyclable catalyst, we carried out many repeats of the recycling experiments to investigate the performance. After the complete reduction of 4-NP, the new nanoreactor was easily separated by centrifugation due to the existence of a silica core with a relatively large density and was redispersed for the next cycle of catalysis. The catalytic reduction of 4-NP was performed at 20 °C for each cycle. As shown in Figure 7, the new nanoreactor could still preserve a higher catalytic efficiency (∼90.2%) till the end of 16 cycles of the recycling experiments, which demonstrated good cycling performance and stability for the new nanoreactor. Therefore, the new nanoreactor turned out to be a promising candidate for catalytical reduction of 4-NP.

## 4. Conclusions

In summary, the AuNPs-based thermoresponsive nanoreactor with high stability for AuNPs and excellent thermosensitivity was successfully synthesized by using PMBA as stabilizer for AuNPs and PNIPAM as a temperature sensitive protect-shell. Such a nanoreactor could have better stability for AuNPs in the gel layer of PMBA, and effectively prevent the aggregation of AuNPs caused by the phase transformation of PNIPAM during the preparation and experimental process. The electron transfer between the N atoms of amide groups in the PMBA and Au atoms of AuNPs played an important role in stabilizing AuNPs with PMBA, which was confirmed by XPS and ATR-FTIR with the interaction nature between AuNPs and PMBA. The newly synthesized nanoreactor was monodispersed and had the characteristics of the good uniformity, regular shape, and nanoscale size, which demonstrated another possibility for the potential applications as chromatography separation, biomedical devices, controlled catalysis, and other areas. The presence of an internal silica core increased the specific gravity of the new nanoreactor for centrifugalization and reutilization. The new nanoreactor with high dispersibility and stability in aqueous solution showed a controllable catalytic activity by altering the reaction temperature, in which it exhibited a higher catalytic activity at 20 °C and 30 °C, but an inhibited catalytic activity at 40 °C for 4-NP. The repeated recycling experiments indicated that the new nanoreactor had a good cycling performance, while still having a preserved high catalytic efficiency for the reduction of 4-NP even after 16 times of recycling. In future applications, the as-synthetized nanoreactor with thermosensitivity, high stability, and good recyclability can be applied in the general catalytic reduction reactions, in particular in exothermic reactions where the reactions are out of control, and also has broad application prospects in the field of green chemistry.

## Supplementary Materials

The following are available online at http://www.mdpi.com/2079-4991/8/12/963/s1, Figure S1: ^1^H-NMR spectra of synthesized microspheres in D_2_O: (**a**) SiO_2_@PMBA microspheres; (**b**) SiO_2_@PMBA@Au microspheres.
