# Supplementary material for: AuNPs-Based Thermoresponsive Nanoreactor as an Efficient Catalyst for the Reduction of 4-Nitrophenol
Source: Nanomaterials (Basel). 2018 Nov 22;8(12):963. doi: 10.3390/nano8120963 (PMC6315678; doi:10.3390/nano8120963)
Supplement: Supplementary file 1 [file nanomaterials-08-00963-s001.pdf]

nanomaterials

Supporting Information

AuNPs-based Thermoresponsive Nanoreactor as an Efficient Catalyst for the Reduction of 4-Nitrophenol

Wei Liu [1,2,3,*], Xiaolian Zhu [2], Chengcheng Xu [2], Zhao Dai [2,*] and Zhaohui Meng [3]

1 State Key Laboratory of Separation Membranes and Membrane Processes/ National Center for International Joint Research on Separation Membranes, Tianjin Polytechnic University, Tianjin 300387, China
2 School of Environmental and Chemical Engineering, Tianjin Polytechnic University, Tianjin, 300387, China; zxl280060217@163.com (X.Z.); 13820360661@163.com (C.X.)
3 Tianjin Colouroad Coatings & Chemicals Co Ltd, Tianjin 300457, China; colouroadmzh@163.com
* Correspondence: liuhuiwen217@yahoo.com or weiliu@tjpu.edu.cn (W.L.); daizhao@gmail.com (Z.D.); Tel.: +86-22-83955660 (W.L.); +86-22-83955621 (Z.D.); Fax: +86-22-83955451 (W.L.)

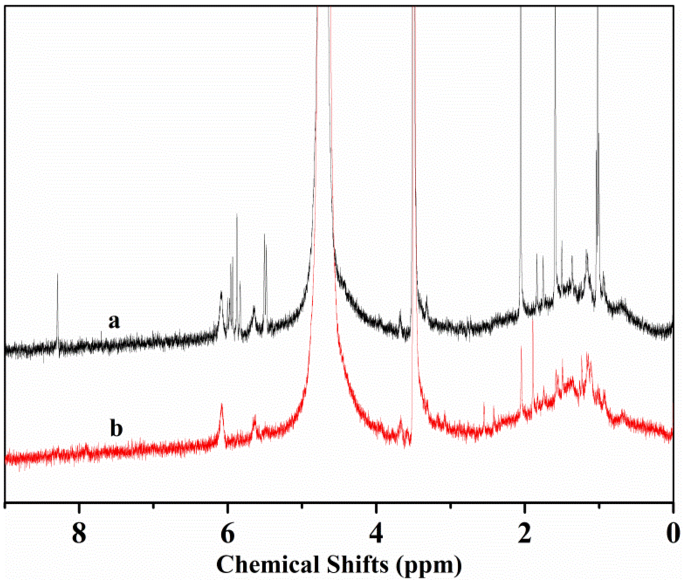

Figure S1. ^{1}H-NMR spectra of synthesized microspheres in D_2O: (**a**) SiO_2@PMBA microspheres; (**b**) SiO_2@PMBA@Au microspheres.

Figure S1 showed the ^{1}H-NMR spectrum of SiO_2@PMBA microspheres and SiO_2@PMBA@Au microspheres. Some changes could be observed by comparing the signals between the two microspheres.

When a ligand is grafted onto the surface of a nanoparticle, its NMR signals are broadened because of the distribution of local environments, which spread the associated chemical shifts, and because of shorter transverse relaxation times, which arise from a decrease of its rotational mobility (degrees of freedom). [1] This broaden can be so significantly that the signal completely flattens out in the baseline, just as disappeared. On the contrary, free functional groups will yield sharp signals. This changes on the NMR spectra have been observed and proved in thiol ligands stabilized AuNPs [2,3]. In this research, the difference could be also observed in the case of amide as the ligands, which could provide evidence for the linkage between Au and N atoms. In curve a, the signal at 8.29 ppm was ascribed to the protons of amide groups (–CONH–) in PMBA, and it significantly flattened out or completely disappeared after immobilizing AuNPs on the SiO_2@PMBA microspheres, (in curve

b), which reflected the functional groups closest to the AuNPs and interacting with AuNPs. Other functional groups closer to AuNPs were methyl and methylene groups on the polymer networks, which corresponded to the sharp signals at 1～2 ppm in curve a. Most of them also disappeared after the immobilization of AuNPs.

In addition, the sharp signals at 5～6 ppm could be assigned to the protons of vinyl groups. Here, the carbon double bonds would came from two parts. One was the residual double bond of the crosslinker without polymerization, which was pended on the polymer chains. The other one was the double bonds produced by disproportionation termination during the polymerization process, which was at the terminal of the polymer chains. The latter was connected with the saturated carbon, resulting in the signals of methylene groups next to these double bonds shifted to high magnetic field. The disappearance of the signals at 5～6 ppm in curve b was due to the vinyl groups simultaneously reduced by $NaBH_4$ with the formation of AuNPs. The stronger signal at 3.46 ppm was attributed to the methylene groups between the two N atoms of MBA, it had no significant changes in curve a and curve b.

References

1. Badia, A.; Demers, L.; Dickinson, L.; Morin, F.G.; Lennox, R.B.; Reven, L. Gold-sulfur interactions in alkylthiol self-assembled monolayers formed on gold nanoparticles studied by solid-state NMR. *J. Am. Chem. Soc.* **1997**, *119*, 11104–11105.
2. Goldmann, C.; Ribot, F.; Peiretti, L.F.; Quaino, P.; Tielens, F.; Sanchez, C.; Chaneac, C.; Portehault, D. Quantified binding scale of competing ligands at the surface of gold nanoparticles: The role of entropy and intermolecular forces. *Small* **2017**, *13*, 1604028.
3. Guo, C.C.; Yarger, J.L. Characterizing gold nanoparticles by NMR spectroscopy. *Magn Reson Chem.* **2018**, *56*, 1074–1082.
